# Editorial: Differential activation of cell death pathways in macrophages as a result of adaptation to divergent microenvironment

**DOI:** 10.3389/fimmu.2025.1623587

**Published:** 2025-05-22

**Authors:** Krzysztof Guzik, Philippe Saas, David Masson

**Affiliations:** ^1^ Department of Immunology, Faculty of Biochemistry, Biophysics and Biotechnology, Jagiellonian University, Kraków, Poland; ^2^ Université Grenoble Alpes, Institut national de la santé et de la recherche médicale (INSERM) U1209, CNRS Unité Mixte de Recherche (UMR) 5309, Institut pour l’Avancée des Biosciences (IAB), DIMAC, Grenoble, France; ^3^ Etablissement Français du Sang Auvergne-Rhône-Alpes, R&D Laboratory, Grenoble, France; ^4^ Center for Translational and Molecular Medicine (CTM), Unité Mixte de Recherche (UMR) 1231, Université Bourgogne Europe, Institut national de la santé et de la recherche médicale (INSERM), Dijon, France; ^5^ Laboratoire de Biochimie, CHU Dijon, Dijon, France

**Keywords:** macrophage plasticity, regulated cell death, microenvironmental adaptation, inflammation, single-cell transcriptomics, ferroptosis, atherosclerosis, macrophage

## Introduction

Macrophages are ubiquitous immune cells residing within tissues, where they play critical roles in recognizing, engulfing, and processing foreign particles, dead cells, and debris. They are highly plastic, rapidly adapting their phenotype and functions in response to local microenvironmental signals (1, 2). This plasticity extends to their cell death modalities: while apoptosis typically resolves inflammation and promotes tolerance as well as tissue repair, necroptosis, pyroptosis, and ferroptosis are associated with inflammatory responses of varying intensity (3, 4). Recent advances, notably single-cell technologies, have revealed that the expression and activation of cell death machinery within macrophages are highly context-dependent. Therefore, macrophage death is increasingly recognized as not merely a consequence of injury or dysfunction but also as an active effector mechanism shaping tissue homeostasis and pathologies, as exemplified in atherosclerosis (5–7). This Research Topic aimed to explore the hypothesis that microenvironmental cues modulate the propensity of macrophages to undergo specific cell death pathways, impacting their protective or pathogenic roles across a wide range of diseases. The collected contributions shed light on the diversity of macrophage death responses under various pathological conditions, with a focus on cardiovascular, metabolic, and inflammatory diseases.

## Summary of contributions

### Macrophage death in atherosclerosis

Several contributions highlighted how microenvironmental factors within atherosclerotic plaques influence macrophage death. In the study by Pilot et al., the inhibition of caspase-8 in macrophages shifted cell death from apoptosis to necroptosis. Despite reduced systemic inflammation, this switch led to larger necrotic cores and aggravated plaque burden, underlining the critical role of regulated cell death pathways in plaque stability. The work on Nrf2-deficient macrophages by Sarad et al. revealed how the loss of this antioxidant transcription factor in aortic macrophages promotes an inflammatory phenotype and alters the expression of death-related genes. Single-cell transcriptomic profiling identified specific subpopulations with enhanced susceptibility to ferroptosis and DNA damage, suggesting that iron homeostasis and oxidative stress are pivotal modulators of macrophage death in early atherogenesis. In a comprehensive review, Neels et al., examined the interplay between macrophage death and plaque calcification. Beyond the failure of efferocytosis, apoptotic bodies and other cell death forms such as necroptosis and pyroptosis were shown to act as nucleation sites for calcium phosphate deposition, contributing to plaque instability.

### Macrophage adaptation in other pathologies

Other contributions expanded this theme of macrophage death in other pathological settings. In diabetic cardiomyopathy, Zhang et al. reviewed the role of macrophages in the progression of cardiac dysfunction. They emphasized how prolonged exposure to metabolic stress reshapes macrophage phenotypes and potentially their death responses, highlighting emerging therapeutic avenues targeting macrophage plasticity. Using a model of radiation-induced heart injury, Cao et al. performed single-cell RNA sequencing to track macrophage dynamics during early tissue damage and repair. They identified stage-specific macrophage subsets with distinct death and survival profiles, suggesting that macrophage plasticity in the injured heart is tightly orchestrated to balance inflammation and tissue remodeling.

### Mechanistic insights into macrophage cell death

At the mechanistic level, the study of HV1 proton channel inhibition by Kovacs et al. demonstrated how interference with proton homeostasis in macrophages leads to pH imbalance, ceramide accumulation, and reduced viability in a polarization-dependent manner. These findings underscore how ion channel regulation intricately connects to macrophage survival and death, particularly in inflammatory contexts. Finally, a review on the interplay between pyroptosis, necroptosis, and ferroptosis by Makuch et al. synthesizes current knowledge on the interdependence of these pathways. The authors proposed that macrophages dynamically balance different forms of regulated cell death in response to microenvironmental challenges, shaping outcomes in diseases such as atherosclerosis and cancer. Macrophage death is the ultimate effector mechanism responding to distinct stimuli of the microenvironment. Different types of necrotic death exhibit diverse dynamics and are differently controlled. The manuscripts of Pilot et al., and Makuch et al. illustrate this differential feature of necroptosis and ferroptosis. In an oxidized low-density lipoprotein-rich environment, inhibition of caspase-8 promotes necroptosis. Whereas in a Fe^2+^-rich environment, the Fenton reaction triggers macrophage ferroptosis. The Fenton reaction occurs with a rapid rate—typical for inorganic chemistry—and is not biologically controlled in contrast to caspase-8 activity. Only the instantaneous balance between hydroxyl radicals and intracellular antioxidants determines macrophage ferroptosis or survival.

### Conclusion and Perspectives

Overall, the contributions to this Research Topic illustrate the remarkable sensitivity of macrophages to their tissue environment, particularly regarding the activation of death pathways. The modulation of macrophage death by environmental factors not only affects local immune responses but also plays a decisive role in disease progression and tissue integrity. Future research should aim to better characterize macrophage subsets that are prone to specific types of death, identify molecular signatures associated with these processes, and develop therapeutic strategies targeting macrophage death or survival in a disease-specific manner. Understanding macrophage death as an active and regulated phenomenon opens new possibilities for therapeutic innovation across many fields. Finally, several important themes emerge: the role of controlled apoptosis in preserving tissue integrity; the damaging effects of inflammatory forms of death like necroptosis and ferroptosis; and the importance of redox signaling, ion homeostasis, and epigenetic mechanisms in determining macrophage fate.
